# Longitudinal Imaging of T Cells and Inflammatory Demyelination in a Preclinical Model of Multiple Sclerosis Using ^18^F-FAraG PET and MRI

**DOI:** 10.2967/jnumed.120.259325

**Published:** 2022-01

**Authors:** Caroline Guglielmetti, Jelena Levi, Tony L. Huynh, Brice Tiret, Joseph Blecha, Ryan Tang, Henry VanBrocklin, Myriam M. Chaumeil

**Affiliations:** 1 Department of Physical Therapy and Rehabilitation Science, University of California, San Francisco, San Francisco, California;; 2 Department of Radiology and Biomedical Imaging, University of California, San Francisco, San Francisco, California;; 3 CellSight Technologies, Inc., San Francisco, California

**Keywords:** T cells, multiple sclerosis, central nervous system, ^18^F-FAraG PET imaging, MRI

## Abstract

Lymphocytes and innate immune cells are key drivers of multiple sclerosis (MS) and are the main target of MS disease-modifying therapies (DMT). Ex vivo analyses of MS lesions have revealed cellular heterogeneity and variable T cell levels, which may have important implications for patient stratification and choice of DMT. Although MRI has proven valuable to monitor DMT efficacy, its lack of specificity for cellular subtypes highlights the need for complementary methods to improve lesion characterization. Here, we evaluated the potential of 2′-deoxy-2′-^18^F-fluoro-9-β-d-arabinofuranosylguanine (^18^F-FAraG) PET imaging to noninvasively assess infiltrating T cells and to provide, in combination with MRI, a novel tool to determine lesion types. **Methods:** We used a novel MS mouse model that combines cuprizone and experimental autoimmune encephalomyelitis to reproducibly induce 2 brain inflammatory lesion types, differentiated by their T cell content. ^18^F-FAraG PET imaging, T2-weighted MRI, and T1-weighted contrast-enhanced MRI were performed before disease induction, during demyelination with high levels of innate immune cells, and after T cell infiltration. Fingolimod immunotherapy was used to evaluate the ability of PET and MRI to detect therapy response. Ex vivo immunofluorescence analyses for T cells, microglia/macrophages, myelin, and blood–brain barrier (BBB) integrity were performed to validate the in vivo findings. **Results:** ^18^F-FAraG signal was significantly increased in the brain and spinal cord at the time point of T cell infiltration. ^18^F-FAraG signal from white matter (corpus callosum) and gray matter (cortex, hippocampus) further correlated with T cell density. T2-weighted MRI detected white matter lesions independently of T cells. T1-weighted contrast-enhanced MRI indicated BBB disruption at the time point of T cell infiltration. Fingolimod treatment prevented motor deficits and decreased T cell and microglia/macrophage levels. In agreement, ^18^F-FAraG signal was decreased in the brain and spinal cord of fingolimod-treated mice; T1-weighted contrast-enhanced MRI revealed intact BBB, whereas T2-weighted MRI findings remained unchanged. **Conclusion:** The combination of MRI and ^18^F-FAraG PET enables detection of inflammatory demyelination and T cell infiltration in an MS mouse model, providing a new way to evaluate lesion heterogeneity during disease progression and after DMT. On clinical translation, these methods hold great potential for stratifying patients, monitoring MS progression, and determining therapy responses.

## Visual Abstract

**Figure:**
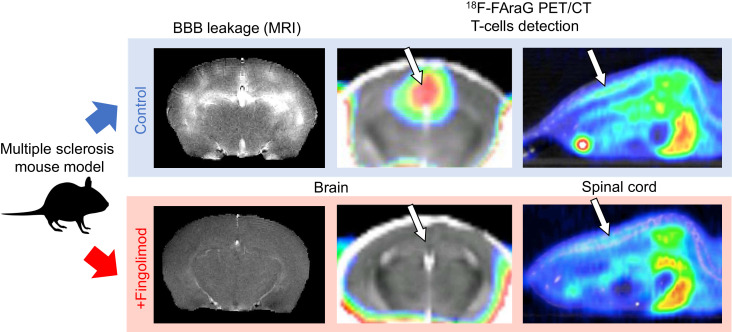

Adaptive and innate immune cells play a critical role in the onset and progression of multiple sclerosis (MS) (*1*). Currently, all disease-modifying therapies (DMT) that slow MS progression act on the immune system (*2*). Evidence from histopathology analysis of brain tissue indicates that lesions are heterogeneous and that T cell concentrations are highly variable at sites of active demyelination (*3*–*7*). Although conventional MRI is well established to diagnose and monitor MS progression by detecting lesions, its inability to provide specific information on underlying cellular events justifies the development of new complementary tools to improve lesion characterization (*8*,*9*). Briefly, T1-weighted contrast-enhanced MRI and T2-weighted MRI can assess blood–brain barrier (BBB) integrity and demyelination or inflammation, respectively, but are unable to inform on immune cell type presence (*1*,*9*). Imaging of immune cells would improve our understanding of lesion progression across the course of MS and, most importantly, allow for proper stratification of patients and optimized choice of therapeutic regimen.

Recently, molecular imaging using novel tracers for PET have enabled the in situ detection of T cells in living organisms. On entry to the cell through nucleoside transporters, 2′-deoxy-2′-^18^F-fluoro-9-β-d-arabinofuranosylguanine (^18^F-FAraG) is phosphorylated by cytoplasmic deoxycytidine and mitochondrial deoxyguanosine kinases (*10*), trapping this PET probe in the cell. ^18^F-FAraG PET has shown high specificity for activated T cells in vitro and in models of graft-versus-host disease, inflammatory arthritis, and cancer (*10*–*14*) and is currently under investigation in clinical trials. Because MS lesions may differ in their T cell accumulation, ^18^F-FAraG PET appears as a complementary method to assess lesion types in MS.

The novel model of cuprizone and experimental autoimmune encephalomyelitis (EAE) for MS (*15*) presents 2 types of brain lesions temporally distinct and highly reproducible. First, cuprizone administration induces demyelination and elevated microglia/macrophages. Next, after EAE immunization, these lesions additionally display T cell infiltration.

Here, we investigated the potential of ^18^F-FAraG PET to detect T cell infiltration into the central nervous system (CNS) in the cuprizone-EAE model of MS. We also evaluated the ability of ^18^F-FAraG PET to detect response to fingolimod, a DMT that limits the infiltration of T cells into the CNS (*16*). Our findings showed that MRI allowed the detection of inflammatory demyelination independently of T cell presence, whereas ^18^F-FAraG PET enabled lesion stratification by informing on T cell load, thus providing a novel methodology to evaluate immune cell heterogeneity in lesions.

## MATERIALS AND METHODS

### Animals and Experimental Design

All animal research was approved by the Institutional Animal Care and Use Committee of the University of California, San Francisco. The cuprizone-EAE model was induced by feeding 8-wk-old C57BL/6J female mice (*n* = 32, Jackson Laboratories) a cuprizone-supplemented diet (0.25%, Sigma) for 3 wk, followed by 2 wk of normal chow. At the start of the sixth week, the mice were immunized with myelin oligodendrocyte glycoprotein 35–55 in complete Freund adjuvant. The mice received an intraperitoneal injection of pertussis toxin on the day of immunization and the following day (Hooke Laboratories). After immunization, the mice were evaluated daily for motor symptoms and were scored using an EAE scale as previously described (*17*). A first group of control mice underwent a 60-min dynamic PET acquisition (*n* = 4) immediately followed by ex vivo biodistribution studies to evaluate tracer distribution and accumulation. A second group of cuprizone-EAE mice (*n* = 10) underwent longitudinal PET and MRI sessions at the following time points: before disease induction (baseline), after 3 wk of cuprizone (W3), and at the end of the seventh week, which corresponds to 14 ± 1 d postimmunization (W7–14dpi). Euthanasia was performed after the last imaging session. A third group of cuprizone-EAE mice (*n* = 10) received a daily administration of a fingolimod solution by oral gavage (0.3 mg/kg; Combi-Blocks) for 14 ± 1 d after immunization, starting on the day of immunization. PET and MRI were performed at W7–14dpi and immediately followed by euthanasia. A fourth group of mice that did not undergo any imaging procedures was used for histologic analyses (*n* = 4 control, *n* = 4 W3 cuprizone). The experimental outline is shown in Supplemental Figure 1A (supplemental materials are available at http://jnm.snmjournals.org).

### MRI Acquisition

All in vivo MR experiments were conducted on a 14.1-T vertical MR system (Agilent Technologies) equipped with 100 G/cm gradients and a ^1^H volume coil (inner diameter, 40 mm). For each imaging session, the mice were anesthetized using isoflurane (1.5%–2% in O_2_) and a 27-gauge catheter was placed in the tail vein for injection of the gadolinium contrast agent (gadobutrol [Gadavist; Bayer]). The animals were placed in a water-heated cradle with the head secured to ensure similar positioning between experiments. Respiration was continuously monitored using PC-SAM software (SA Instruments, Inc.). T2-weighted MRI was performed using the following parameters: an echo time/repetition time of 11.8/3,000 ms, a rapid-imaging-with-refocused-echoes factor of 8, a slice thickness of 0.5 mm, a gap of 0.25 mm, 6 averages, a matrix of 256 × 256, and a field of view of 30 × 30 mm. T1-weighted MRI was acquired before and 2 min after injection of a gadobutrol bolus (1 mmol/kg of body weight) using an echo time/repetition time of 2.09/120 ms, a slice thickness of 0.8 mm, a gap of 0.2 mm, 10 averages, a matrix of 256 × 256, and a field of view of 20 × 20 mm.

### PET Acquisition

All in vivo PET/CT scans were performed on a dedicated small-animal PET/CT scanner (Inveon; Siemens Healthcare). ^18^F-FAraG was synthesized as previously described (*11*). The mice were anesthetized using isoflurane (2% in O_2_), and ^18^F-FAraG (3.7–5.5 MBq in 100–200 μL of saline) was injected into a catheterized tail vein. After ^18^F-FAraG injection, the mice were either imaged immediately for 60 min or were allowed to recover from anesthesia and be ambulatory for 60 min to permit tracer distribution, and whole-body static scans (a 15-min PET acquisition followed by a CT scan for anatomic reference and attenuation correction) were then performed under anesthesia using isoflurane (2% in O_2_) with warming and constant monitoring. PET images were reconstructed using the ordered-subsets expectation maximization algorithm provided by the manufacturer.

### Ex Vivo Biodistribution Studies

Immediately after the dynamic PET acquisitions, control mice (*n* = 4) were euthanized and blood and brain were collected. The blood and brain tissue were weighed and radioactivity measured using a Hidex AMG γ-counter. The percentage injected dose per gram of tissue was calculated against a standard of known activity.

### MRI and PET Analyses

The corpus callosum, hippocampus, and cortex were manually delineated on T2-weighted images using AMIRA software (Thermo Fisher), as indicated in Supplemental Figure 1B. Normalized mean T2-weighted signal intensity for each region of interest was calculated as previously described (*18*). Whole-brain alteration of the BBB was assessed by manually delineating hyperintense regions on contrast-enhanced T1-weighted images as shown in Supplemental Figure 1C. To investigate regional differences in the PET signal throughout the brain, we coregistered PET/CT images with T2-weighted MR images using VivoQuant software (version 4.0, patch 3; Invicro). Partial-volume correction was not performed. Regions of interest were manually delineated over the entire brain, corpus callosum, hippocampus, and cortex on T2-weighted images, and corresponding mean PET signal values, expressed as percentage injected dose per gram of tissue, were calculated for each region of interest. In addition, we manually delineated the cervical/thoracic and lumbar spinal cord segments as described by James et al. (*19*), and the subiliac lymph nodes, to obtain the corresponding mean percentage injected dose per gram of tissue (Supplemental Fig. 1D).

### Immunofluorescence Analyses

All analyses were performed as previously described (*17*), except for the inclusion of CD3 and fibrinogen immunostaining using the following combinations: rabbit anti-fibrinogen (A0080, 1:200 dilution; Dako) with donkey anti-rabbit Alexa Fluor 555 (A31572, 1:600 dilution; Invitrogen); rat anti-CD3 (MCA500G, 1:400 dilution; BioRad) with goat anti-rat Alexa Fluor 488 (A11006, 1:800 dilution; Invitrogen). Quantitative analyses of immunofluorescence images were performed using NIH ImageJ (version 1.52p). CD3 cell number was manually evaluated using the cell counter tool from multiple regions depicted in Supplemental Figure 1E. Macrophages/microglia (ionized calcium binding adaptor molecule 1 [Iba1]), myelin basic protein (MBP), and fibrinogen levels were determined on the basis of image-covering staining and expressed as percentage of total area (*17*).

### Statistical Analyses

Data are reported as mean ± SE. In the figures, dots represent the mean value obtained for each individual and lines indicate longitudinal measurements. Statistical analyses of longitudinal in vivo PET and MRI were performed using a mixed-effect model with the restricted maximum-likelihood method (GraphPad Prism, version 8.4.3). One-way ANOVA was used to assess the statistical significance of the MBP, Iba1, CD3, and fibrinogen immunofluorescence staining. Correlation analyses were performed using the Pearson correlation coefficient. *P* values were corrected for multiple testing using the Tukey post hoc test.

## RESULTS

### ^18^F-FAraG PET and MRI Detect T Cells and Inflammatory Demyelination in the Cuprizone-EAE Mouse Brain

**FIGURE 1.**
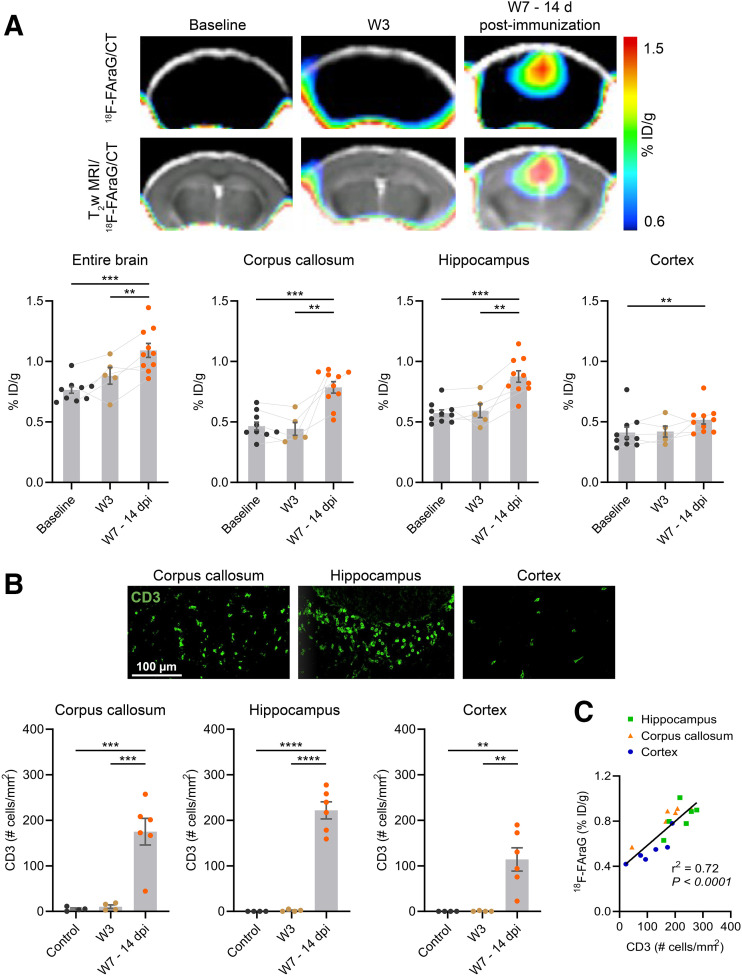
(A) ^18^F-FAraG PET/CT images and ^18^F-FAraG PET/CT overlaid on T2-weighted (T_2_w) MR images at baseline, W3, and W7–14dpi. Graphs show ^18^F-FAraG uptake in entire brain, corpus callosum, hippocampus, and cortex. (B) Immunofluorescence images of CD3 T cells (green) from corpus callosum, hippocampus, and cortex at W7–14dpi. Quantification of CD3 T cells in corpus callosum, hippocampus, and cortex at baseline, W3, and W7–14dpi. (C) Correlation of ^18^F-FAraG signal with CD3 T cells at W7–14dpi. ID = injected dose. **P ≤ 0.01. ***P ≤ 0.001. ****P ≤ 0.0001.

Dynamic ^18^F-FAraG PET acquisitions in control animals revealed a steady increase in ^18^F-FAraG signal in the brain over 60 min. The brain-to-blood ratio calculated from PET images (over the last 10 min) was 1.1 ± 0.2, and the brain-to-blood ratio calculated from subsequent ex vivo biodistribution studies was 0.73 ± 0.1 (Supplemental Fig. 2). These results indicate that ^18^F-FAraG accumulates in the brain and crosses the intact BBB in mice. Next, we evaluated the potential of ^18^F-FAraG PET to detect brain-infiltrating T cells in the cuprizone-EAE model. Figure 1A shows PET/CT images obtained from the mouse brain, and a clear increase in ^18^F-FAraG signal can be seen in the corpus callosum and hippocampal area at W7–14dpi. Quantitative analyses revealed that ^18^F-FAraG signal was significantly increased in the entire brain after immunization at W7–14dpi (*P* = 0.0004 and *P* = 0.038 compared with baseline and W3, respectively). A significant ^18^F-FAraG signal increase at W7–14dpi was also seen in the corpus callosum (*P* = 0.0003 and *P* = 0.0097 compared with baseline and W3, respectively) and hippocampus (*P* = 0.0005 and *P* = 0.007 compared with baseline and W3, respectively). A higher ^18^F-FAraG signal was found in the somatosensory cortex (*P* = 0.0077 compared with baseline). Histologic analyses confirmed a significant increase in CD3 T cells after immunization at W7–14dpi, with the highest CD3 T cell density in the hippocampus (*P* < 0.0001 compared with both baseline and W3), followed by the corpus callosum (*P* = 0.0006 and *P* = 0.0007 compared with baseline and W3, respectively) and cortex (*P* = 0.004 and *P* = 0.0041 compared with baseline and W3, respectively), in line with the PET imaging results (Fig. 1B). Also in line with the imaging data, T cell levels were not significantly increased in any region at W3 (Supplemental Fig. 3). Analysis revealed a significant positive correlation between ^18^F-FAraG uptake and CD3 T cell number (Fig. 1C, *r*^2^ = 0.72, *P* < 0.0001).

**FIGURE 2.**
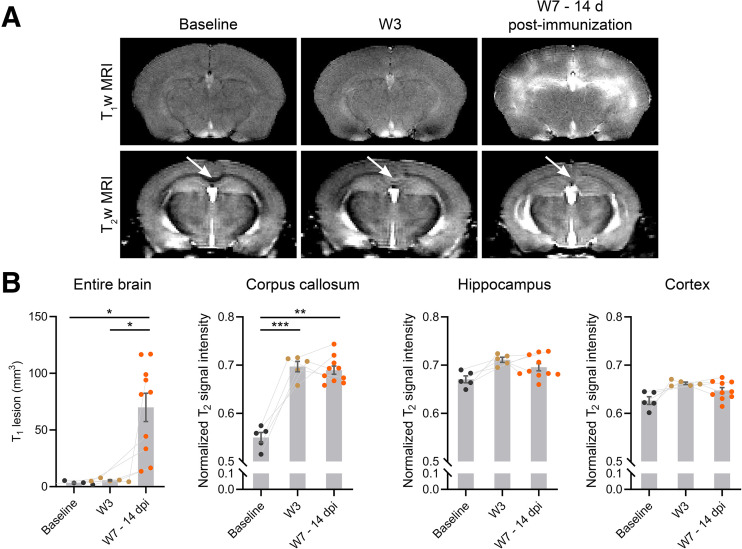
(A) T1-weighted (T_1_w) MR images after injection of gadolinium and T2-weighted (T_2_w) MR images at baseline, W3, and W7–14dpi. Arrows indicate corpus callosum. (B) Corresponding quantification of T1-enhancing lesions and normalized T2-weighted signal intensity from corpus callosum, hippocampus, and cortex. *P ≤ 0.05. **P ≤ 0.01. ***P ≤ 0.001.

Next, we assessed the potential of MRI to detect brain alteration at W3 and W7–14dpi during inflammatory demyelination. A leaky BBB was observed after immunization (W7–14dpi) (Fig. 2, *P* = 0.0418 and *P* = 0.05 compared with baseline and W3, respectively). The volume of T1-enhancing lesion varied greatly among animals, ranging from 13 to 117 mm^3^. T2-weighted MRI detected the presence of hyperintense lesions in the corpus callosum, but not in the hippocampus or cortex, after the cuprizone diet (W3, *P* = 0.0009) and immunization (W7–14dpi, *P* = 0.0011).

**FIGURE 3.**
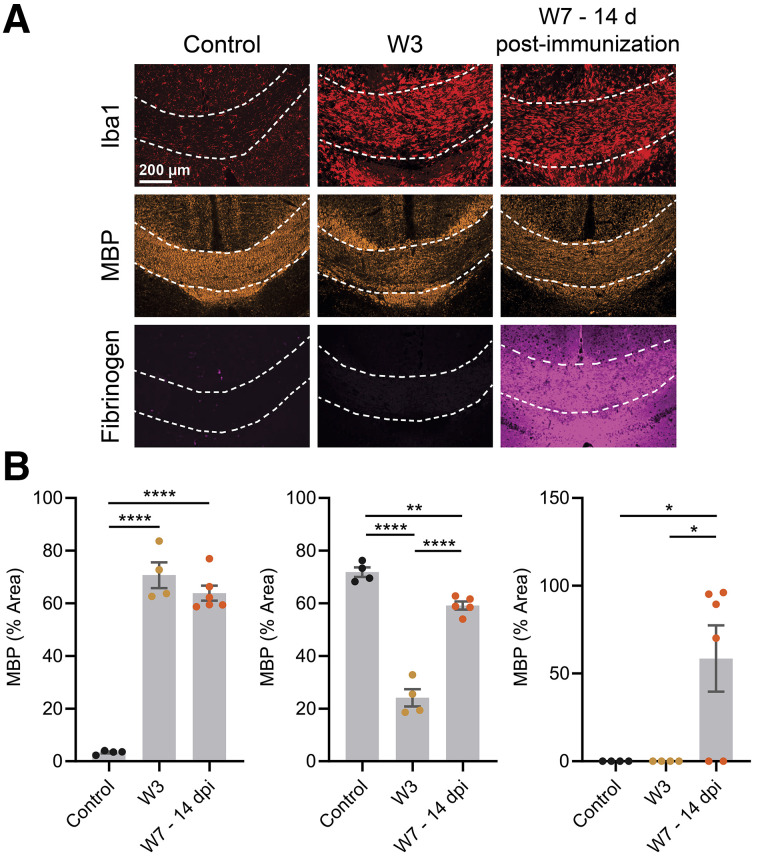
(A) Immunofluorescence images from corpus callosum (dashed lines) for microglia/macrophages (Iba1, red), myelin (MBP, orange), and fibrinogen (magenta). (B) Corresponding quantitative analyses in control animals, at W3 and W7–14dpi. *P ≤ 0.05. **P ≤ 0.01. ****P ≤ 0.0001.

Inflammatory demyelination at W3 and W7–14dpi was confirmed by immunofluorescence analysis. Innate immune cells (Iba1) were significantly increased at W3 (*P* < 0.0001) and W7–14dpi (*P* < 0.0001) in the corpus callosum, along with demyelination (MBP) at W3 (*P* < 0.0001) and W7–14dpi (*P* = 0.0059) (Fig. 3). Fibrinogen staining confirmed BBB disruption at W7–14dpi (*P* = 0.0353 compared with both baseline and W3). Similarly, an increase in innate immune cells (Iba1) was observed at W3 and W7–14dpi in the hippocampus (*P* = 0.0309 and *P* = 0.0002 compared with baseline) and at W7–14dpi in the cortex (*P* < 0.0001 compared with baseline) (Supplemental Fig. 4). Interestingly, demyelination (MBP) was present at W3 in the hippocampus (*P* = 0.0117 compared with baseline) and W7–14dpi in the cortex (*P* = 0.0048 and *P* = 0.0158, compared with baseline and W3, respectively). Fibrinogen staining confirmed BBB breakdown in the hippocampus at W7–14dpi (*P* = 0.0004 and *P* = 0.0002 compared with baseline and W3, respectively).

### ^18^F-FAraG PET Detects T Cells in the Spinal Cord and Lymph Nodes

**FIGURE 4.**
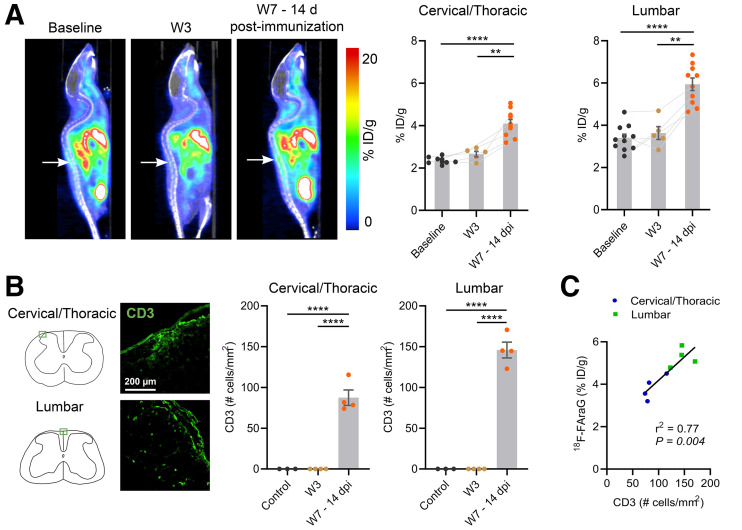
(A) ^18^F-FAraG PET/CT sagittal images at baseline, W3, and W7–14dpi. Arrows point to lumbar spinal cord. Graphs show corresponding quantification of ^18^F-FAraG signal in cervical/thoracic and lumbar spinal cord. (B) Immunofluorescence images of CD3 T cells (green) in cervical/thoracic and lumbar spinal cord at W7–14dpi, and quantification of CD3 immunostaining at baseline, W3, and W7–14dpi. (C) Correlation of ^18^F-FAraG signal with CD3 T cells at W7–14dpi. ID = injected dose. **P ≤ 0.01. ****P ≤ 0.0001.

**FIGURE 5.**
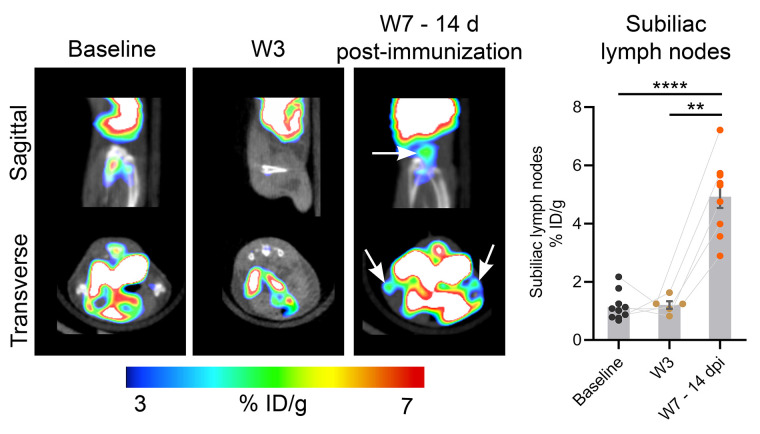
^18^F-FAraG PET/CT images showing subiliac lymph nodes and quantification of ^18^F-FAraG uptake at baseline, W3, and W7–14dpi. ID = injected dose. **P ≤ 0.01. ****P ≤ 0.0001.

After immunization, the mice showed phenotypical symptoms of EAE pathology indicative of spinal cord lesions (mean EAE score, 2.2 ± 0.3 at W7–14dpi). As shown in Figure 4A, a clear ^18^F-FAraG signal arising from the lumbar spinal cord can be observed at W7–14dpi. Quantitative analyses revealed a significant increase in ^18^F-FAraG signal in both the cervical/thoracic (*P* < 0.0001 and *P* = 0.0081 compared with baseline and W3, respectively) and the lumbar (*P* < 0.0001 and *P* = 0.0068 compared with baseline and W3, respectively) spinal cord at W7–14dpi. CD3 T cells were detected at W7–14dpi in the cervical/thoracic (*P* < 0.0001 and *P* < 0.0001 compared with baseline and W3, respectively) and the lumbar (*P* < 0.0001 and *P* < 0.0001 compared with baseline and W3, respectively) spinal cord (Fig. 4B) and significantly correlated with ^18^F-FAraG signal (Fig. 4C, *r*^2^ = 0.78, *P* = 0.004). ^18^F-FAraG signal was also increased in the subiliac lymph nodes at W7–14dpi (*P* < 0.0001 and *P* = 0.0084 compared with baseline and W3, respectively), in agreement with the elicited adaptive immune response after immunization (Fig. 5).

### ^18^F-FAraG PET Detects Response to Fingolimod Therapy

We evaluated whether ^18^F-FAraG could detect the effect of fingolimod treatment. Untreated animals showed weakness or paralysis of the limbs (mean EAE score at W7–14dpi, 2.2 ± 0.3). In contrast, fingolimod-treated mice did not show any sign of EAE pathology (mean EAE score at W7–14dpi, 0 ± 0), confirming the effect of fingolimod treatment.

**FIGURE 6.**
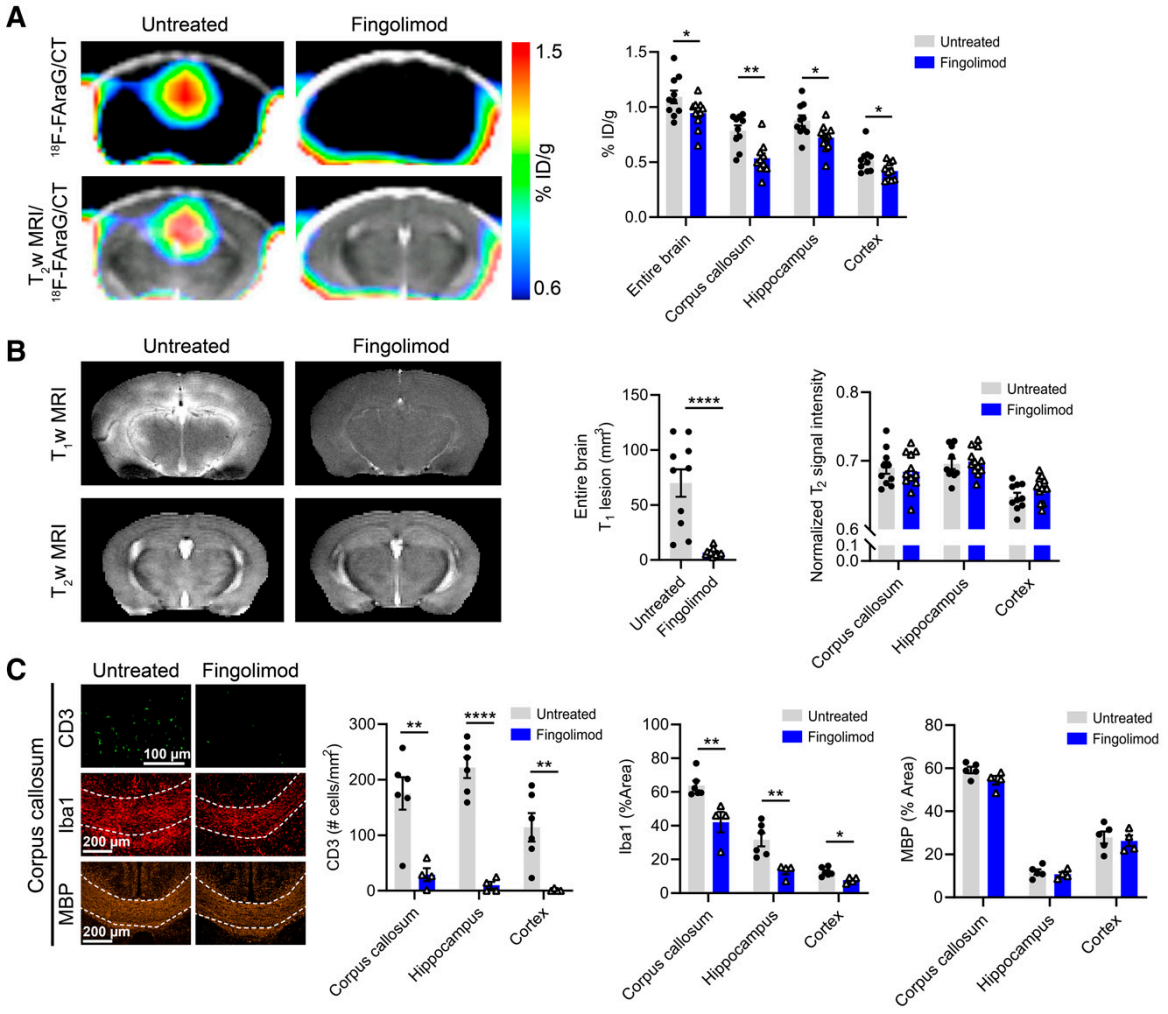
(A) ^18^F-FAraG PET/CT and ^18^F-FAraG PET/CT overlaid on T2-weighted (T_2_w) MR images from untreated and fingolimod-treated animals at W7–14dpi. Graph shows quantification of ^18^F-FAraG signal in entire brain, corpus callosum, hippocampus, and cortex. (B) T1-weighted (T_1_w) and T2-weighted MR images of fingolimod-treated and untreated mice and corresponding quantification of T1-enhancing lesions and normalized T2-weighted signal intensity of corpus callosum, hippocampus and cortex. (C) Immunofluorescence images of corpus callosum for CD3 T cells (green), microglia/macrophages (Iba1, red), and myelin (MBP, orange) from untreated and fingolimod-treated mice at W7–14dpi. Graphs show quantification of immunofluorescence images from corpus callosum, hippocampus, and cortex. ID = injected dose. *P ≤ 0.05. **P ≤ 0.01. ****P ≤ 0.0001.

^18^F-FAraG signal was significantly lower in the entire brain (*P* = 0.046*7*), corpus callosum (*P* = 0.0011), hippocampus (*P* = 0.0169), and cortex (*P* = 0.0355) of fingolimod-treated animals (Fig. 6A). No T1-enhancing lesions could be detected in the brain of fingolimod-treated mice (*P* < 0.0001, Fig. 6B). Interestingly, T2-weighted MRI did not detect any differences between fingolimod-treated and untreated mice. Histologic analyses confirmed a significantly lower CD3 T cell number in the corpus callosum (*P* = 0.0047), hippocampus (*P* < 0.0001), and cortex (*P* = 0.0077) after fingolimod treatment (Fig. 6C, Supplemental Fig. 5). Microglia and macrophage levels (Iba1) were decreased in fingolimod-treated mice, whereas demyelination (MBP) was comparable between fingolimod-treated and untreated animals. Furthermore, ^18^F-FAraG signal was significantly lower in the spinal cord and lymph nodes of fingolimod-treated mice (Supplemental Fig. 6).

## DISCUSSION

A way to noninvasively visualize immune cells within the CNS would tremendously help improve the diagnosis and monitoring of neurodegenerative disorders, particularly MS. Here, we showed the high potential of ^18^F-FAraG PET in the detection of T cell infiltration and accumulation within the CNS and lymphoid tissue in a MS model, and we demonstrated its ability to detect response to an immunomodulatory DMT.

The cuprizone-EAE model was particularly suited for this study because it allows evaluation of 2 lesion types well separated in time (W3 and W7–14dpi) within the same animal. Furthermore, in space, the differential load of T cells throughout the brain, with different levels in white and gray matter, enables evaluation of the capability of ^18^F-FAraG PET to detect various levels of infiltrating T cells. We showed that ^18^F-FAraG signal was strongly associated with T cell density in the brain and spinal cord. Prior in vitro studies have shown that ^18^F-FAraG uptake also occurs in macrophages, but the agent is not retained because of low levels of cytoplasmic deoxycytidine and deoxyguanosine kinases (*10*). This is likely the case in our study, because high level of microglia/macrophages are observed after a cuprizone diet (W3) (*18*), but ^18^F-FAraG signal remained unchanged compared with baseline.

In healthy conditions, the adult brain has a limited capacity for de novo synthesis of nucleosides and relies on uptake through nucleoside transporters present on the BBB, thus providing a means for ^18^F-FAraG delivery to the brain (*20*). Although a low background signal of ^18^F-FAraG in the brain enables detection of small changes in tracer accumulation (*11*), we noted a higher ^18^F-FaraG signal in ventral brain areas (Supplemental Fig. 7). ^18^F-FAraG brain uptake in the 2 cohorts of control mice showed different values (Fig. 1; Supplemental Fig. 2), as might be explained by different anesthesia lengths or acquisition parameters (static vs. dynamic). When ^18^F-FAraG brain uptake was normalized to ^18^F-FAraG uptake in muscle, no significant differences between these cohorts were found (Supplemental Fig. 8).

In cuprizone-EAE, the increase in ^18^F-FAraG signal coincided with the time point of BBB disruption measured by contrast-enhanced T1-weighted MRI and fibrinogen immunostaining—a timing that may facilitate ^18^F-FAraG accumulation in the brain. However, we showed in Supplemental Figure 9 that T1-enhancing lesion volumes did not correlate with ^18^F-FAraG signal and that ^18^F-FAraG signal was increased in non–T1-enhancing lesions containing high levels of CD3 T cells. These findings further support that ^18^F-FAraG provides additional information with regard to T cell accumulation. Discrepancies between imaging modalities might also be explained by differences in molecular weight and lipid solubility of the different agents used to evaluate BBB permeability (*21*), although fibrinogen and gadobutrol are well established. Additionally, small lesions might remain undetected because of a low MRI resolution and the partial-volume effect.

Recent work by Chen et al. described 1-(2′-deoxy-2′-^18^F-fluoroarabinofuranosyl) cytosine (^18^F-FAC) use in the EAE model (*22*). ^18^F-FAC has been shown to accumulate in leukocytes through cytoplasmic deoxycytidine kinase phosphorylation in models of immune activation (*23*,*24*) and accumulated in nearly equal amounts within brain-infiltrating T cells and innate immune cells. When compared with our study, ^18^F-FAC and ^18^F-FAraG showed a similar magnitude of change, displaying a 180% and 140% increase in brain PET signal compared with control mice, respectively, although the MS models were different. Similarly, after fingolimod therapy, ^18^F-FAC accumulation was decreased by 37% and ^18^F-FAraG accumulation by 32% in the corpus callosum. Although fingolimod blocks egress of lymphocytes out of secondary lymphoid organs (*16*), we observed lower ^18^F-FAraG uptake in lymph nodes. This finding may be explained by fingolimod’s inhibition of T cell activation (*25*) and will require further investigation in future studies. Surprisingly, in contrast to our findings, ^18^F-FAC did not accumulate in the spinal cord, despite high T cell levels and macrophage infiltration (*26*). This result was explained by low spinal cord cytoplasmic deoxycytidine kinase expression and thus suggests that ^18^F-FAraG might provide a more accurate detection of T cells because of its higher affinity for deoxyguanosine kinase. Additionally, ^18^F-FAC is deaminated in humans, limiting its clinical translation. In contrast, ^18^F-FAraG has shown feasibility and safety in humans (*11*) and is under investigation in multiple clinical trials.

Alongside PET, conventional MRI proved essential to detect brain lesions. T2-weighted MRI detected white matter lesions, independently of their T cell content (W3 and W7–14dpi). However, this method failed to detect fingolimod therapy response, as both demyelination and immune cell content influence the T2 tissue properties and are important components of lesions before and after DMT (*27*). A major drawback of T2-weighted MRI is the low sensitivity for gray matter lesion detection (*18*,*28*). In contrast, ^18^F-FAraG PET detected changes in white and gray matter and thus could help identify lesions invisible on T2-weighted MRI (Figs. 1–2 and 6). T1-weighted contrast-enhanced MRI indicated BBB alterations after EAE immunization, as previously described (*29*), and detected fingolimod efficacy, because no BBB disruption was observed in treated animals, supporting fingolimod’s action on endothelial cells (*30*).

## CONCLUSION

Altogether, our results indicate that ^18^F-FAraG PET can be used to noninvasively image T cell infiltration within the brain and spinal cord during a demyelinating event and detects response to immunomodulatory therapy. These data support further investigation of ^18^F-FAraG as a potential agent for clinical translation, with the aim of improving lesion characterization in MS patients and provide a novel tool to evaluate response to therapies.

## DISCLOSURE

This work was supported by research grants NIH R21AI153749 and NIH R01NS102156, by NMSS research grant RG-1701-26630, by the Hilton Foundation–Marilyn Hilton Award for Innovation in MS Research (17319), and by the Dana Foundation’s David Mahoney Neuroimaging Program. Jelena Levi is employed by CellSight Technologies. CellSight Technologies is commercializing ^18^F-FAraG as a PET tracer for evaluation of immune response in immunotherapy. No other potential conflict of interest relevant to this article was reported.

## Notes

KEY POINTS **QUESTION:** Can ^18^F-FAraG PET imaging detect T cell infiltration in the CNS in a preclinical model of MS and improve lesion characterization when combined with conventional MRI? **PERTINENT FINDINGS:** ^18^F-FAraG uptake increased in the brain and spinal cord of an MS mouse model and correlated with T cell density, providing additional information on T cell presence in MRI-detected lesions and enabling lesion stratification. After treatment with an immunomodulatory DMT, ^18^F-FAraG uptake was decreased in the brain and spinal cord, highlighting its potential to monitor response to immunotherapy. **IMPLICATIONS FOR PATIENT CARE:** ^18^F-FAraG may provide a new methodology to evaluate T cell presence in the CNS and thus may enable patient stratification and monitoring of immune cell dynamics during disease progression and after therapy in MS and other T cell–mediated neurologic conditions.
